# Correction: Bax and Bak can localize to the endoplasmic reticulum to initiate apoptosis

**DOI:** 10.1083/jcb.20030208401202026c

**Published:** 2026-02-05

**Authors:** Wei-Xing Zong, Chi Li, Georgia Hatzivassiliou, Tullia Lindsten, Qian-Chun Yu, Junying Yuan, Craig B. Thompson

Vol. 162, No. 1 | https://doi.org/10.1083/jcb.200302084 | July 7, 2003

The authors have discovered that the publisher inadvertently introduced an error in Fig. 2 A. The graph in the center (Bax/MCF7) was mistakenly duplicated in the right column of the second row (Bax/293T). The original and corrected Fig. 2 are shown here. This error does not affect the conclusions of the study, and the figure legend remains unchanged.

This error has been corrected online but remains in print and in the PDF. The publisher apologizes for any confusion this may have caused.

**Figure:**
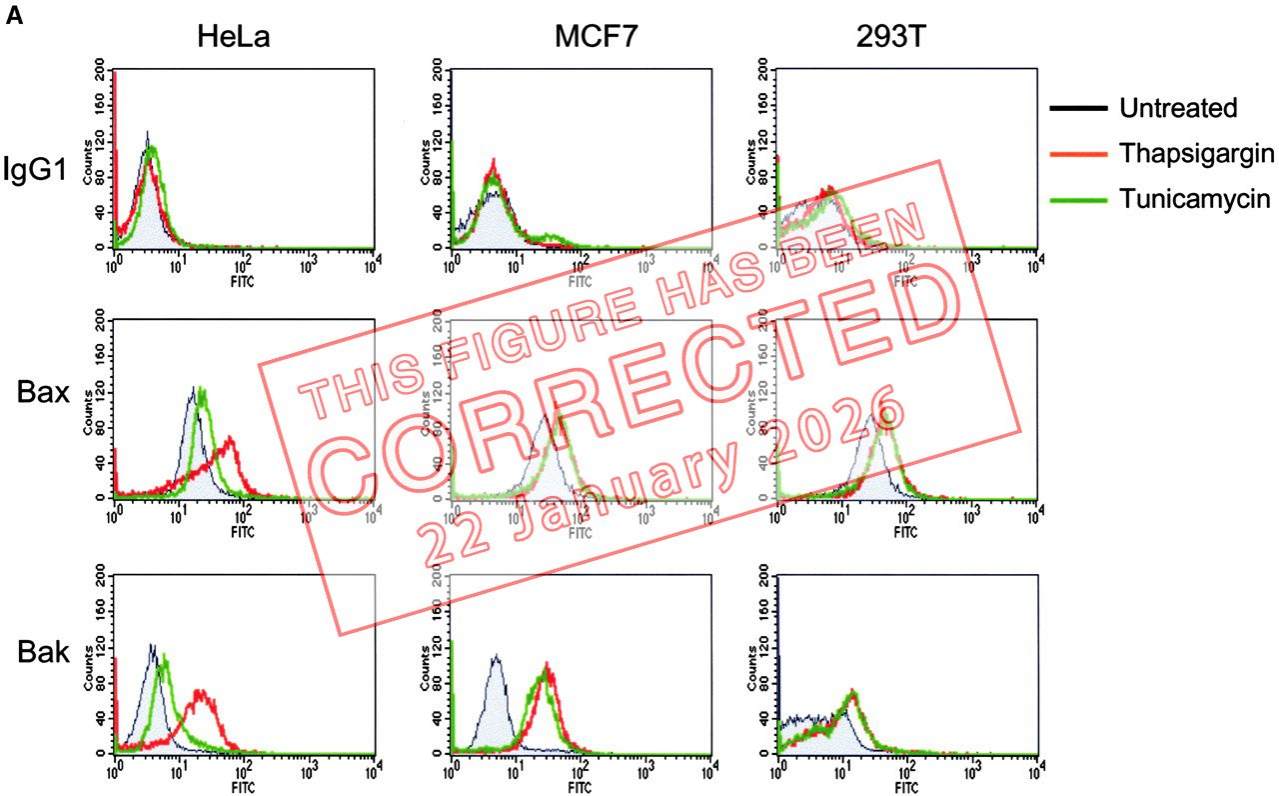

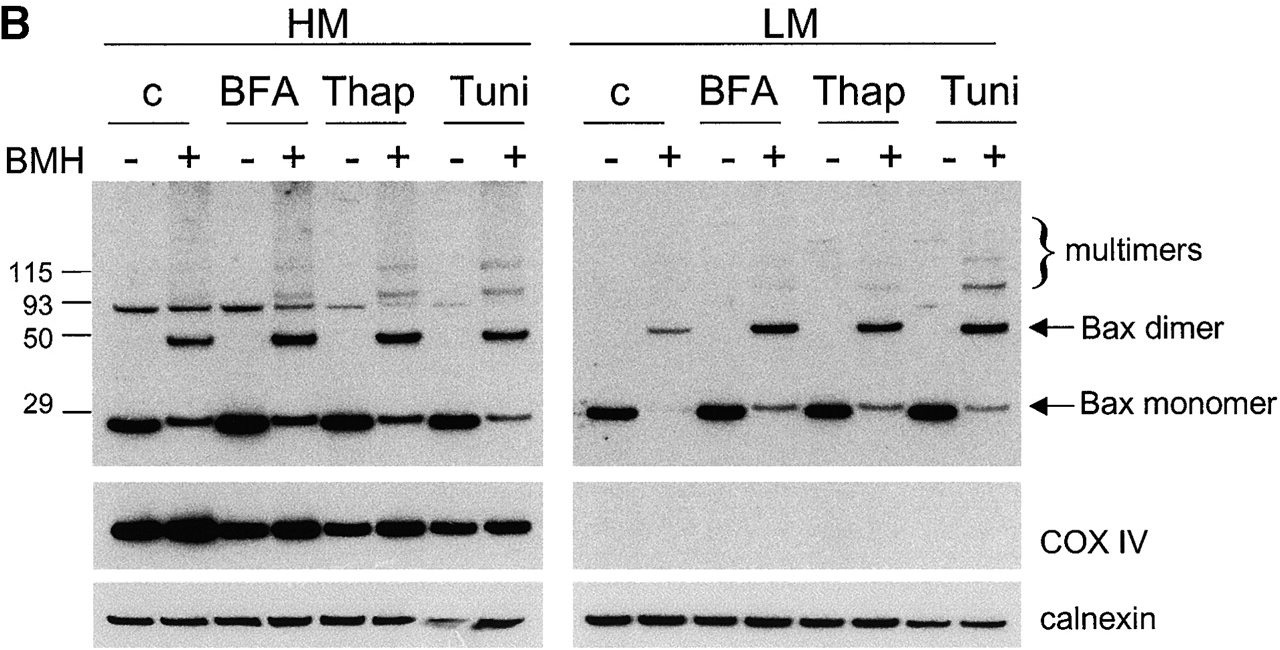


**Figure 2.**
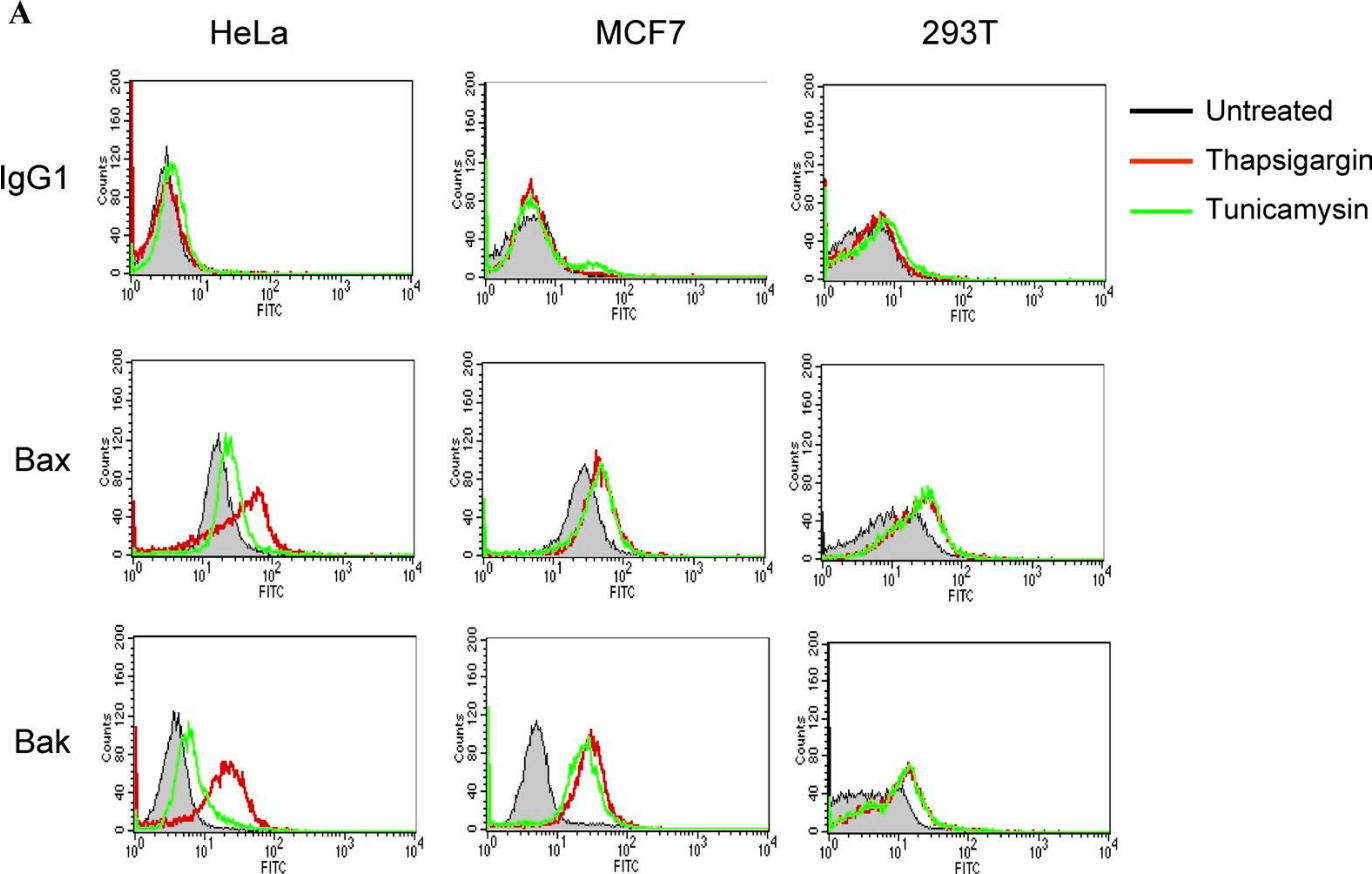

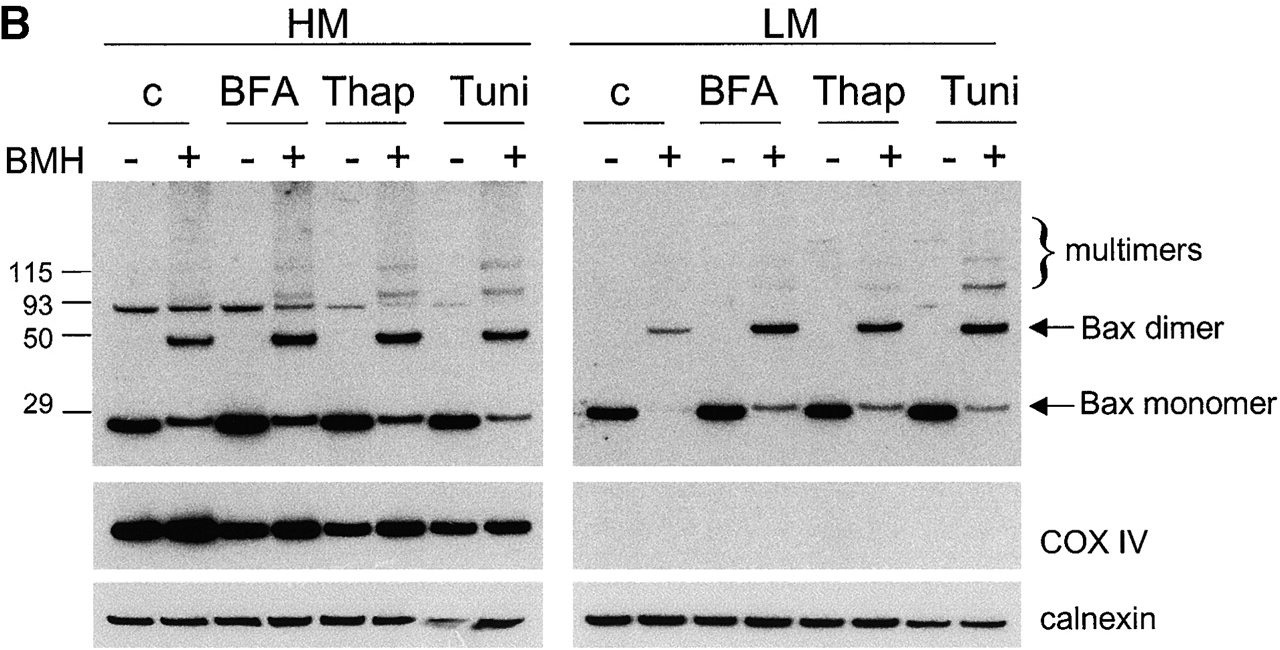
**ER stress induces Bax and Bak conformational changes and oligomerization at the ER.** (A) ER stresses induce the conformational changes of Bax and Bak. HeLa, MCF7, and 293T cells were treated with thapsigargin (Thap; 2 µM) or tunicamycin (Tuni; 5 µg/ml) for 36 h. Cells were fixed in 0.25% paraformaldehyde in PBS for 5 min. Cells were incubated with a control antibody (mouse IgG1) and conformation-sensitive antibodies against Bax or Bak, followed by incubation with FITC-conjugated secondary antibody. (B) ER stress induces Bax oligomerization at the ER. Wild-type MEFs were treated with brefeldin A (BFA; 10 µg/ml), Thap (2 µM), or Tuni (10 µg/ml) for 24 h. Cells were resuspended in hypotonic buffer A and disrupted. 5 mM BMH cross-linking reagent was added to cross-link the oligomerized proteins. Cells were subjected to subcellular fractionation to obtain the HM and LM fractions. 20 µg of total protein was separated on a 4–12% gradient NuPAGE gel. A polyclonal anti-Bax antibody was used to detect Bax. COX IV and calnexin are shown as indicators of the purity of the fractionation and as loading controls.

